# Arthropods and inherited bacteria: from counting the symbionts to understanding how symbionts count

**DOI:** 10.1186/1741-7007-11-45

**Published:** 2013-04-15

**Authors:** Olivier Duron, Gregory DD Hurst

**Affiliations:** 1 Institut des Sciences de l'Evolution, Centre National de la Recherche Scientifique (CNRS), Université Montpellier 2, 34095 Montpellier Cedex 05, France; 2 Institute of Integrative Biology, University of Liverpool, Liverpool L69 7ZB, UK

## 

Before 1990, the existence of heritable microbes in insects was recognized only by specialists working in the field of symbiosis. In the mid-1990s, the advent of simple PCR assays led to the widespread appreciation of one particular symbiont, *Wolbachia*. A deeper investigation of the biodiversity of symbionts led to a third phase of knowledge: bacteria from many different clades have evolved to be heritable symbionts, typically transmitted maternally and thought not to be routinely horizontally (infectiously) transmitted. In an issue of *BMC Biology *published in 2008, we observed that a diverse assemblage of maternally inherited bacteria were present in a broad range of arthropods [1]. Whilst *Wolbachia *remained the dominant bacterium, we noted that three other inherited bacteria, *Spiroplasma*, *Cardinium *and *Arsenophonus*, were also common. Overall, 33% of arthropod species examined were observed to carry at least one of these four symbionts.

It is now clear that many more than one-third of species carry heritable symbionts. Any sampling regime produces 'false negatives', species that are infected but where infection is not detected. This occurs when infected individuals go unsampled, either because the symbiont is present in a minority of individuals in the population, or where the sample is from an uninfected population but individuals from other areas in the species range are infected. Further, surveys such as ours looked for particular bacteria, and ignored clades of bacteria that are restricted to particular host groups. Altogether, it is now clear that the majority of arthropod species carry inherited microbes, and that these microbes are diverse (Figure 1).

**Figure 1:**
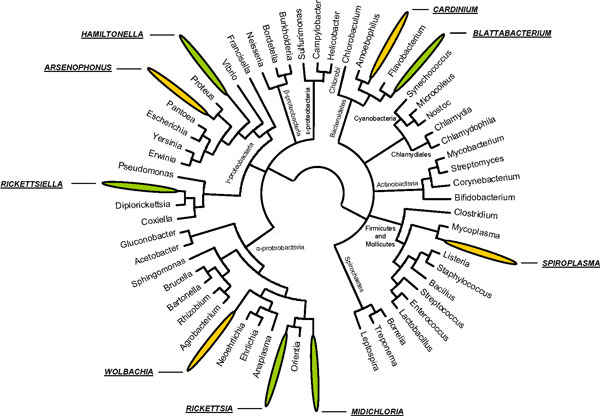
**Evolutionary relationships of heritable bacteria found in arthropods (not exhaustive)**. Yellow, globally common heritable bacteria; green, rare heritable bacteria.

In this piece we review two aspects of the biology of heritable symbionts where our views have changed substantially in the last five years. First, we note that the effect of infection on a host is more complex than previously considered. Symbionts increase host fitness more commonly than previously believed, and they may also have multiple impacts on their host. Second, whilst it has long been established that symbionts transfer from one host species to another, it was previously considered that these horizontal transfer events were rare. We now understand that some symbionts transfer very frequently between species. Further, symbiont genes transfer into the host nucleus, host genes transfer into the symbiont, and symbionts may also acquire genes from other symbionts. Thus, there are complex webs of genetic information exchange.

## Most symbionts are actually beneficial, but not essential, and many have multiple impacts on their host

In our paper in 2008, we started from the premise that the bacteria we were studying were parasites of arthropod reproductive systems that spread using sex ratio distortion or cytoplasmic incompatibility as drive mechanisms. However, we noted that, in most cases, the nature of the interactions between these inherited bacteria (including *Wolbachia*) and their hosts was not known, and thus remained to be determined.

It is now clear that the heritable microorganisms we studied are not simply reproductive parasites. *Wolbachia *in arthropods has emerged as a conditional mutualist conferring advantages under certain environmental conditions. For instance,

*Wolbachia *increases fecundity of *Drosophila melanogaster *reared on iron-restricted or -overloaded diets, and can thus confer a direct fitness benefit during periods of nutritional stress [2]. The most dramatic findings are that *Wolbachia *can protect their hosts against attack by natural enemies. *Wolbachia *infection interferes with the replication and transmission of a wide range of pathogens and parasites (including RNA viruses, bacteria, protozoa and nematodes), and protects its host from parasite-induced mortality [3]. These properties have led to *Wolbachia *being developed as an agent to limit transmission of human pathogens by arthropod vectors [4].

*Wolbachia *is not alone in being a defensive symbiont. Diverse symbionts in aphids provide protection against parasitic wasp and fungal attack, and include members of the *Rickettsia *and *Spiroplasma *genera [5,6]. *Drosophila*, an organism intensively studied with respect to determinants of resistance to parasites, was recently revealed to have defensive *Spiroplasma *[7]. *Paederus *rove beetles carry heritable *Pseudomonas *that produces a toxin that deters predators [8]. Apart from protective effects, symbionts mediate variation in heat tolerance, plant use and body color [5,9]. For instance, the symbiont *Rickettsiella *changes the body color of the aphid host from red to green, and is thus likely to influence relative susceptibility to predators [9].

It is also now clear that individual symbionts have multiple properties. For instance, the *Wolbachia *strain in *D. melanogaster *was characterized initially as one producing only weak reproductive manipulation but is now also known to confer nutritional and protective benefits. Similarly, Himler *et al. *[10] found that *Rickettsia *infection in whiteflies both increased host survival and reproductive success, and biased the sex ratio towards production of daughters, a classical feature of reproductive parasites. The further observation that some symbiont effects are revealed only in novel hosts suggests multiple potencies may be common [11]. Multiple effects on the host are also very important in the application of heritable microbes in disease control. The reproductive parasitism of *Wolbachia *allows it to invade and be maintained at high frequency in a population, such that the effect it has on the competence of individuals to act as disease-carrying vectors is then observed at a population scale [4].

## The complex web by which heritable symbionts move between hosts and genes move between symbionts and from symbiont to host

Phylogenetic evidence indicates that most symbioses originate following horizontal transfer of an existing symbiont from one host species to another [12,13]. It was always presumed that horizontal transfer events were rare, occurring on evolutionary rather than ecological timescales. However, heritable symbionts have been shown to combine inheritance with infectious transmission within, and sometimes between, species in a number of cases (Table 1). Ecological connections, such as feeding on a shared plant host, are major drivers for these rapid movements of symbionts across insect communities. Symbiont transfer between individuals of different host species will be an important determinant of the global incidence of infection. Further, the rate of horizontal transfer in some of these systems is such that a single host/single symbiont framework may be insufficient for understanding the population and evolutionary dynamics in some symbiotic systems.

**Table 1:** Case studies where heritable bacteria commonly transfer horizontally on an ecological timescale

Bacterium	Movement occurs between	Ecological context	References
***Arsenophonus nasoniae***	Parasitic wasp species	Sharing of host pupa	[19]
***A. phytopathogenicus***	Species of phytophagous *Hemiptera *	Through plant phloem	[20]
***Rickettsia***	*Bemisia *whiteflies	Through plant phloem	[21]
***Wolbachia***	Parasitic wasp species	Sharing of host egg	[22]
***Hamiltonella defensa***	Aphids	Via exterior of the ovipositor of parasitic wasps	[23]

Movement of symbionts and the traits they encode are now known to be very common through both inheritance and horizontal transfer. It is also beginning to emerge that other genetic connections are possible. Transfer of symbiont genetic information to the host's nuclear genome is known to occur frequently, although the functional significance of transferred material is less clear [14]. Symbionts can also exchange genetic information with other symbionts. Bacteria are, of course, promiscuous with respect to DNA, and different symbionts commonly reside within the same host cell, providing the opportunity for gene transfer. There is strong evidence *Wolbachia *exchange phage when two strains co-infect a host [15]. What is yet to be established is the extent of gene exchange between different heritable symbionts, and whether this leads to the transfer of traits such as natural enemy resistance. Comparisons of the genomes of *Cardinium *and *Wolbachia *strains inducing cytoplasmic incompatibility suggested many common mechanisms inherent in intracellular symbiosis between these very divergent bacteria, but no evidence that this was associated with gene exchange [16]. Nevertheless, phage can shuttle genes from one heritable bacterium species to another. *Arsenophonus *and *Hamiltonella *share a common phage, implying either direct transfer of the phage, or indirect transfer of genes within the phage through recombination of different phage elements [17]. Given that phage presence can determine the capacity of *Hamiltonella *to protect its aphid host against parasitoid wasp attack [18], phage transfers like this may move important traits between symbiont taxa.

## Concluding remarks

Our understanding of the nature of host-inherited symbiont interactions has advanced since the advent of PCR led to the widespread discovery of *Wolbachia *and its domination of the literature. The case studies above demonstrate the importance of a diversity of symbionts as a source of evolutionary innovation in insects: symbionts alter host phenotype, and because they are heritable form part of host adaptation. Perhaps the most remarkable observation of recent time is the speed of symbiont-associated adaptation. Within 30 years, a *Spiroplasma *strain has invaded many North American populations of *D. neotestacea*, driven by the protection it provides against a parasitic nematode [7]. In whiteflies, an inherited *Rickettsia *strain that enhances offspring number and survival has spread from less than 1% of individuals infected to 97% in only 6 years [10]. Rapid spread of beneficial symbiont-encoded traits may be commonplace in insects. In this context, horizontal transfer through ecological interactions can serve as an immediate and powerful mechanism of rapid adaptation. The mutational source of adaptation - a symbiont in other members of the ecological community rather than a mutation of existing genetic material - is likely to change our understanding of arthropod evolution.

## Note

This article is part of the *BMC Biology *tenth anniversary series. Other articles in this series can be found at http://www.biomedcentral.com/bmcbiol/series/tenthanniversary.
